# A Case of Uterine Hydatids

**Published:** 1847-01

**Authors:** Francis H. Gordon

**Affiliations:** Clinton College, Tennessee


ART. II.—*A Case of Uterine Hydatids.* By FRANCIS H. GORDON, of Clinton College, Tennessee.

Mrs. A. G., æt. fifty-one, has had ten children, the youngest being over six years old. During the spring of 1845 her catemania became irregular, and generally persisted only about two days. The abdomen soon began to enlarge, and her friends thought she was again pregnant; but each supposed menstrual period caused temporary diminution of the abdomen. About the 25th December the discharge ceased, and did not return till 21st March, 1846. During this interval the abdomen enlarged rapidly, and by the last named date was as large as at the full term of uterogestation. Now, the discharge, which appeared to be serum, sometimes slightly tinged with blood, showed itself, and continued to return every two or three days, but in so moderate a quantity as not materially to affect the patient's strength or the size of the abdomen. About the 20th of April, it became profuse, consisting largely of blood, and caused speedy prostration. The recumbent posture and a liberal use of alum were the means resorted to by the family; and the woman soon recovered so as to be up. After this, the discharge, which still returned every few days, assumed the appearance of a mixture of pus, blood, and serum. The woman's health gradually declined. Feet generally cold; brain and nervous system, liver, stomach and bowels, all suffered.

*June* 15, 1846. I was called to see her in the night, and found her with strong labor pains, the fundus of the uterus being about an inch above the umbilicus; the whole organ was hard and quite sore. The woman had daily had smart pains for more than a week, attended with the expulsion of lumps, which I supposed to be coagula. With the view of suspending or moderating the uterine action till the organ could be got into a safer condition for expelling its contents, I immediately gave a grain of acetate of morphia, and directed a warm poultice to be laid over the whole abdomen. These means produced partial ease for about five hours, when the paroxysms returned with violence, and in about one hour expelled a mass of *hydatids* amounting to nearly a chamber pot full, followed by profuse hemorrhage and dangerous prostration. The uterus was grasped at the fundus so as to cause reduction. I then gave four grains of acetate of lead. The hemorrhage moderated, and the strength of the patient rallied in three hours, and with the reaction the pains returned. Fearing the result of another scene so perilous as the one lately witnessed, I gave a grain and a half of the acetate of morphia, which induced several hours sleep. At 4 o'clock, P. M., on the 16th, a mass of hydatids was expelled, weighing several ounces, which was shown to the patient at her request; she remarked that it was just such as had been discharged almost daily for more than a week, some of them being less and others larger.

A broad bandage was now firmly and smoothly applied; small and oft repeated doses of sulph. and carb. magnesia were directed as an aperient; patient to be kept cool, quiet and recumbent; poultices over the abdomen to be resorted to often enough to keep down all signs of inflammation; daily injections of warm water into the vagina, and diet light but nutricious. From this time to the 24th, parcels of hydatids were daily expelled, but the pains were moderate and the hemorrhage inconsiderable. Febrile symptoms underwent gradual abatement, and improvement was progressive. At this time, 24th October, the woman's health appears to be perfect.

I have carefully preserved the mass of hydatids expelled during my first visit; it being a most interesting specimen. I suppose the number of cysts it contains to be more than a thousand, varying from the size of a millet seed up to that of a plum. Their matrix, or medium of attachment to the uterus, consists in part of coagulating lymph infiltrated with blood; but a larger portion much resembles the maternal placenta. The necks of the primary, or parent cysts, penetrate deeply into this substance; secondary hydatids are attached to the sides and summits of these; tertiary ones to the secondary, and so on, in some instances to the sixth generation. Their most common shape is that of a Florence flask; but some are cylindrical and connected at their extremities by pedicles. They are of two colors, a large proportion being perfectly transparent, and the remainder, perhaps one-fifth, varying from a pale red up to the deep hue of a ripe cherry.

Could the contents of the uterine cavity have been viewed in its unbroken arrangement, these must have presented a curious and interesting picture. The whole internal surface was lined with what may be fitly termed a placental mould, for the nourishment of a most luxuriant vegetation. As if nature had been unmindful of her common laws, and disposed to frolic upon a human constitution, in imitating some splendid grotto, the whole surface of the cavity was studded, in rich profusion, with fruits of all grades, from those which had just dropped their petals, up to those of full growth and maturity; between many of which were insinuated filamentous folds resembling leaflets. But not contented with the most dense production the surface could contain, racemes were made to spring from the sides and summits of the primary fruits, and from these other clusters were pendant, and so on, multiplying in geometrical progression, until the whole cavern was decorated with clusters of various sizes, colors, and grades of maturity; and, to render the process complete, the whole was watered by an intermitting fountain, ever gentle and limpid, save when reddened by the wine of ripe fruits, which, anon, swelled it to a desolating torrent.

October, 1846.

